# Accuracy of fatal occupational injury registration in a high-income country: A comparison of two-source capture-recapture estimates with the number of cases identified in four register systems in Norway, 2000–2003

**DOI:** 10.1016/j.gloepi.2022.100072

**Published:** 2022-03-02

**Authors:** Finn Gjertsen, Johan Lund, Ebba Wergeland

**Affiliations:** a Norwegian Institute of Public Health, Norway; b Retired from the Norwegian Directorate of Health, Norway; c Retired from the Norwegian Labour Inspection Authority, Norway

**Keywords:** Fatal occupational injury, Work-related accidents, Administrative data, Capture-recapture methods, Labour inspection authority

## Abstract

**Background:**

Globally, work-related deaths (injuries and diseases) are a major social and public health problem. Register data on fatal occupational injuries in high-income countries may be considered to have high quality, especially when reporting is mandatory and regulated by law. We aimed to assess the accuracy of work-related injury death statistics in Norway, with reference to the Labour Inspection Authority and three other on-going registration systems (the cause-specific mortality register, the register for governmental compensations, and the register for insurance companies).

**Methods:**

In this register-based study, we used the capture-recapture technique to adjust for undercounting. We investigated whether the capture-recapture method using two or three sources gave a valid estimate of fatal occupational injuries as compared with the number of cases identified in four registers administrated by the Norwegian Labour Inspection Authority, Statistics Norway, the Labour and Welfare Administration, and Finance Norway. The inclusion criteria were fatal unintentional injuries among residents of Norway between 2000 and 2003 that occurred while working for income in private and public land-based industries. We obtained ethical and legal approvals.

**Results:**

In a period of four years (2000−2003), the Labour Inspection Authority registered 171 occupational injury deaths among residents employed in land-based industries. Two combinations of data sources gave capture-recapture estimates of 246 [95% CI 216; 279] and 265 [95% CI 234; 299] deaths. In total, 246 cases were identified in the four registration systems, which was 44% higher than the number of deaths registered by the Labour Inspection Authority. The Labour Inspection Authority had the most complete register out of the four registration systems.

**Conclusions:**

The capture-recapture method used on two overlapping data sources gave highly valid estimates of the total deaths. We demonstrated the existence of significant weaknesses in the registration systems in a country considered to have high-quality register data.

## Highlights

• Statistics from the Norwegian Labour Inspection Authority under-reported fatal occupational injuries.
• The Labour Inspection Authority had the most complete register of the four registration systems.
• The actual work injury mortality rate was 27.6 per 1 million employed, which is 44% higher than the official rate of 19.2.
• Capture-recapture models gave estimates close to the total number of cases identified in the four registration systems.

## Introduction

Work-related deaths are a major social and public health problem with great potential for prevention. However, the size of the global burden of work-related mortality is hard to determine. Information is missing in a large number of countries, and the tasks of defining, identifying, and counting work-related deaths are not straightforward, especially regarding deaths from work-related diseases [1]. Nevertheless, several research projects have presented estimates of work-related deaths at the global level [1], and recently, Hämäläinen et al. [2] presented an updated estimate of 2.78 million work-related deaths: diseases (2.4 million) were the main contributors, and the remaining 380,500 deaths (14%) were due to injuries.

It has become increasingly common to use administrative and other secondary register data in epidemiological studies [3,4]. Doing so provides easy access to data and often enhances the feasibility of linking personal information (microdata) from multiple data sources. However, easy access to a linked dataset does not necessarily mean that the available information is reliable and produces correct results. In modern research, the concern that study design and setting produce research findings that are false has increased [5]. In this context, evidence regarding the quality of secondary data sources is of importance, especially in high-income countries considered to have a high quality of register data, such as Norway.

The concept of work-related or occupational injuries is usually better defined and easier to identify than work-related or occupational diseases [1,2]. However, this does not mean that the observed differences in mortality from occupational injuries across countries reflect real differences [1,2,[6], [7], [8], [9]]. Diversity and changes in the methods used in the compilation of national statistics may disrupt comparability. In Europe, national statistics on fatal occupational injuries are, in general, based on information that employers are obliged to report to labour inspection authorities according to laws and regulations. Another possible source is the medical death certificate and the cause of death statistics. In countries such as England, France, Germany, Italy, Luxembourg, Norway, Portugal and the United States, a separate item on the death certificate asks the medical certifier whether the death resulted from an injury sustained at work [7,[10], [11], [12]]. The latter is considered by several countries to be too unreliable as a source for compiling statistics or surveillance, compared with statistics from labour inspection authorities.

The completeness of official statistics may be assessed on an aggregated level using two simple methods. The first is to compare the total number of cases from two different registration systems that are presumed to count the same incidents. Another is to use one data source and compare statistics from the last available year with figures from previous years [13]. If two independent reporting systems show approximately the same aggregated number, it may be used as an indicator of a high degree of completeness (i.e., high reliability). Small variations in the total numbers may be expected due to minor differences in definition, law, and classification systems. Similarly, when comparing the most updated statistics with figures from previous years, relatively small variations may indicate a valid count, and the differences could be explained by a mix of random variations and substantial changes. If the total number is small, maybe less than 100, the random variations between years tend to be relatively large [10,14]. Nevertheless, an assumption about high completeness based on a comparison of aggregated data is not necessarily correct. Coverage and reporting errors may exist. There might be cases in the target population that are not registered, and among the registered, there might be cases that are incorrectly included [3]. A study of deaths due to active tuberculosis recorded in two data systems (the tuberculosis and mortality registers) in Norway between 1977 and 1989 showed good correspondence between the two sources on an aggregated level and suggested that the total number in the official mortality statistics was fairly correct [15]. The comparison of microdata showed that one third of the tuberculosis deaths in the mortality register were underreported and one third were overreported, using the tuberculosis register as a reference [15]. Another study that examined two surveillance systems for occupational injury deaths in the United States between 1992 and 1997 showed essential differences in the figures; however, both systems reported similar patterns for demographics, industry, occupation, and type of incident [16].

In two previous papers, we investigated the completeness and quality of fatal work-related injury data in different registration systems [17,18]. Using microdata, we showed that many cases registered by the governmental Labour Inspection Authority were not registered as work injuries in the mortality register in Statistics Norway [17]. We then collected information from two other data sources: the Labour and Welfare Administration (compensation for occupational injuries) and Finance Norway (information from private insurance companies); several new cases were identified [18]. The Labour Inspection Authority missed information about deaths in road traffic accidents, in health and social services and in the military. Deaths in the northern part of Norway have also been underreported [17]. The risk pattern for industry groups did not significantly change after the use of supplementary information from the Labour and Welfare Administration and private insurance companies registered by Finance Norway [18].

The aim of the present study was to assess the accuracy of work-related injury death statistics in land-based industries in Norway from 2000 to 2003, with reference to the Labour Inspection Authority and three other ongoing registration systems (the cause-specific mortality register, the register for governmental compensations and the register for insurance companies). We investigated whether the capture–recapture estimates were different from the number of cases identified in the four register systems.

## Methods

### Sources of data

This study covered fatal occupational injuries in the four-year period from 2000 to 2003, and we used individual records from four nationwide sources of data: the Norwegian Directorate of Labour Inspection Authority (the register on reported cases of fatal and serious occupational injury), Statistics Norway (the cause-specific mortality register), the Norwegian Labour and Welfare Administration (the register on public occupational injury compensation) and Finance Norway (the national insurance register on private occupational injury compensation, including insurance companies for public employees, with the short name DAYSY). For the years under study (2000–2003), the national insurance register covered about 80% of the insurance market, and for the high-risk industries, the marked share was lower (about 50%–60%). In the period under study, some high-risk industries used national or international insurance companies that did not send data to Finance Norway; this also applied to governmental employees (Kari S. Mørk, Finance Norway, personal communication 19 January 2015).

The national population register (the National Registry) in the Norwegian Tax Administration was used to identify citizens registered by the Labour Inspection Authority with incomplete or incorrect identification numbers. We also received aggregated information (macrodata) on industry categories from Statistics Norway based on data from regular labour force surveys [19]. Further details are available in our two previous manuscripts [17,18].

### Definitions and exclusions

In general, work-related injuries may be defined by three components: a) injuries that occur in the course of work (workplace injuries), b) injuries that occur in public road traffic incidents in the course of work (work-traffic injuries) and c) injuries that occur whilst travelling to and from work (commuting injuries) [1]. Commuting injuries are difficult to count, and international statistics on work-related injuries are based on the first two elements. Our study did not include commuting injuries, and some refinements were needed regarding the two components “workplace injuries” and “work-traffic injuries”.

The Norwegian Working Environment Act reserves the term “occupational injury” for injuries caused by accidental incidents [20] and obligates the employer to notify the governmental Labour Inspection Authority and the nearest police authorities ‘*if an employee dies or is seriously injured as the result of an occupational accident*’ (cf. §5.2 of the employer's notification obligation) [20], regardless of whether the employee was a resident of Norway or another country. The main exceptions to the act are shipping, and fishing (including processing the catch on board ship), participating in offshore petroleum activities, and engaging in civil and military aviation. These areas are governed by other laws.

Occupational injury deaths registered in the national mortality register were essentially based on information recorded on death certificates. Both the Labour Inspection Authority and the mortality register used the term “occupational accidents” for work-related injuries. The death certificate has a separate section for injuries, including a tick-off item if the death was caused by an occupational accident [21]. In contrast to the Labour Inspection Authority, the mortality register exclusively covered deaths where the decedent was registered as a resident of Norway in the population register at the time of death, whether the death occurred in Norway or abroad [22].

As we compared the registration systems and used a capture-recapture estimator, our definition was limited to injury deaths in land-based industries and activities among residents of Norway. Intentional self-harm (suicide) was not included.

In the four-year period from 2000 to 2003, the Labour Inspection Authority registered 183 occupational accidental deaths; 12 of the decedents were residents of countries other than Norway (Table 1). The remaining 171 deaths were included in our study, and these data were linked with the national mortality register. In total, 162 occupational accidental deaths were registered by Statistics Norway, of which 21 occurred in non-land-based activities (Table 1). These 21 cases were excluded from the analysis.

**Table 1:** Fatal occupational injuries registered in four registration systems and number of cases included in the study. Residents of Norway employed in land-based activities, with rates per 1 million employed persons and 95% confidence intervals (Poisson), 2000–2003.

Data source	Total deaths	Excluded (e.g., non-residents, non-land-based work, disease, suicide)	Included (annual average)
Labour Inspection Authority	183	12	171 (43)
Statistics Norway (mortality register)	162	21	141 (35)
Labour and Welfare Administration	164	6	158 (40)
Finance Norway (insurance companies)^a^	56	6	50 (13)
	Rate	Poisson	
	per 1mill	95% CI	
Labour Inspection Authority	19.2	16.4; 22.3	
Statistics Norway (mortality register)	15.8	13.3; 18.6	
Labour and Welfare Administration	17.7	15.1; 20.7	

a The number from Finance Norway is not directly comparable with the figures in the three other sources, as only a part of all deaths caused by an occupational injury were covered by private insurance systems. Rates not calculated.

Occupational injury deaths registered by the Labour and Welfare Administration and by Finance Norway were cases based on applications for compensation. In total, 41 new incidents were identified, of which 9 occurred in non-land-based activities and were excluded from the analysis. Of the 32 included cases, the Labour and Welfare Administration had registered 27, and Finance Norway had noted 7 (2 of the 32 cases were registered in both registries).

Information from all four data sources was linked. In cases where the information was inconsistent across the data sources, we assessed whether the case should be included as an occupational injury or not. In this assessment, we used cause-of-death information on the death certificate, even in cases where no information about occupational injury was available in the mortality register. We also had separate meetings with the Labour and Welfare Administration and Finance Norway for clarification.

### Lincoln-Petersen method, adjustment for undercounting

Capture-recapture methods are techniques used to estimate the completeness of a register [23,24] when two (or more) overlapping data sources are available. Originally, the capture-recapture methods were developed to estimate the unknown size of a closed wildlife population. The first experiments with the method were conducted by the Dane Carl Georg Johannes Petersen in the 1890s when he estimated the size of a population of plaice (a species of fish) in a fjord (Limfjorden) in Denmark [25]. Since the 1940s, the models have been applied to human populations, and the methods are now used on administrative register data to adjust for undercounting [3,4,23,24].

We used the basic capture-recapture estimator [4,23,24] on two combinations of data sources. Data from the Labour Inspection Authority and Statistics Norway were used in one, and data from the Labour Inspection Authority and Labour and Welfare Administration were used in the other. We did not use data from Finance Norway in the estimator because of the incomplete reporting coverage (see the Sources of data section). By letting the estimated number of occupational injury deaths in the population of residents be N, the number of occupational deaths recorded in the two data sources be N_1_ and N_2_ and the number of fatalities registered in both registers be n_1,2_, the estimator of N is given by

$$\hat{N}=\frac{\;N_{1}x\;N_{2}\;}{n_{1,2}}$$

We performed a three-source capture-recapture estimate (Finance Norway was excluded) using the model in Hook and Regal [26] (p. 248) to assess whether the three-source estimate was significantly different from the two-source estimates.

Regarding the potential dependence between the three data sources, log-linear models, as reviewed in Chao et al. [23] (p. 3132), were applied to the three data sets, with one model based on the assumption of independency among the registers and the other allowing for dependency by including all two-way interactions. For each model, the number of cases that were not recorded in any of the three registration systems was estimated, and whether the model assuming dependency gave a significantly better model fit than the model based on independency was investigated using the likelihood ratio test (deviance test; Ørnulf Borgan, University of Oslo, personal communication 19 March 2021).

A unique 11-digit identification number was used to link records across the data sources. The identification number was assigned to permanent Norway residents by the Norwegian Tax Administration and was used by all the data sources. The cause-of-death registration system, for example, has routinely obtained the ID number from the Tax Administration for all deaths among residents for decades, including deaths where the cause of death information or the death certificate was missing [22]. In a small number of records from the Labour Inspection Authorities, the identification number was incomplete or incorrect. We obtained correct identification numbers for all these cases from the population register in the Tax Authority by using available information (e.g., name, sex, date of birth and municipality of injury). This was feasible due to the low number of cases and allowed us to eliminate doublets in the data and record linkage errors [27].

We used the Poisson mean/rate function in the statistical software Stata/SE version 15.0 to calculate the confidence intervals for the capture-recapture estimates. For the calculation of mortality rates, we used statistics on the number of workers from the national labour force surveys (in total, 8916,800 employed person-years in land-based industries for 2000–2003), received from Statistics Norway [18].

### Ethics

The project was initiated in 2004 (E. Wergeland and F. Gjertsen) and was originally based on approvals from the National Data Inspectorate (concession to handle sensitive microdata) and the Directorate of Health (exemption from the rules of confidentiality). The Norwegian Tax Administration approved the identification of a valid identification number if it was missing or incorrect, and the Regional Committee for Medical and Health Research, South East Norway (references S-07475a, 2010/1177), approved the project. The approvals were later extended regarding the project period, a new member of the project group (J. Lund) and the use of two new data sources (the Labour and Welfare Administration and Finance Norway). We also obtained separate approvals from these two agencies.

## Results

**Fig. 1:**
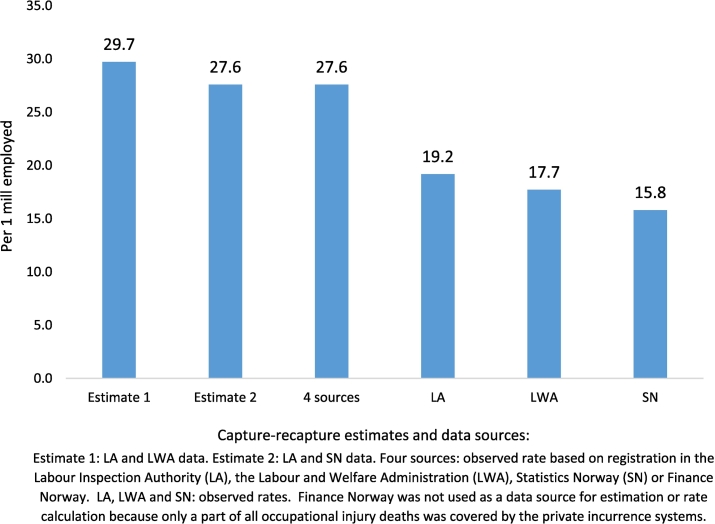
Occupational injury deaths among residents of Norway in land-based activities, 2000–2003. Capture-recapture two-source estimates and cases registered in the data sources; rates per 1 million employed persons.

The estimated true numbers of fatal occupational injuries in land-based activities among Norwegian residents from 2000 to 2003 (based on registrations by the Labour Inspection Authority and the mortality register or the Labour Inspection Authority and the Labour and Welfare Administration) were 246 [95% CI 216; 279] and 265 [95% CI 234; 299], with an annual average of 62 and 66 deaths, respectively. The corresponding annual mortality rates per 1 million employed persons were 27.6 [95% CI 24.2; 31.3] and 29.7 [95% CI 26.2; 33.5] (Fig. 1, Table 2). The three-source estimated rate was 28.1 [95% CI 24.8; 31.9] (Table 2). In the same period, the Labour Inspection Authority registered 171 fatalities (an annual average of 43 deaths), and the corresponding rate was 19.2 [95% CI 16.4; 22.3] per 1 million employed (Table 1). The cause-specific mortality register (Statistics Norway) registered 141 fatalities as occupational injuries, and 98 of these deaths were identified in the register of the Labour Inspection Authority (Table 3). We identified 158 cases in the Labour and Welfare Administration, of which 102 were also registered by the Labour Inspection Authority. In total, 50 cases were identified in the register of Finance Norway, of which 32 were also registered by the Labour Inspection Authority (Table 3). The two-source estimates were 44% ([246/171] – 1 × 100) and 55% ([265/171] – 1 × 100) higher than the number of deaths registered by the Labour Inspection Authority during the four years from 2000 to 2003. Nevertheless, the Labour Inspection Authority had the most complete registration of the four other registration systems.

**Table 2:** Capture-recapture estimate (two and three sources) of occupational injury deaths among residents of Norway employed in land-based activities. Rates per 1 million employed persons and 95% CI (Poisson), 2000–2003.

Data sources^a^	Capture-recapture estimate	95% CI	Employed (in 1000)	Rate (per 1 mill)	95% CI (rate)
LA and SN	246	216; 279	8916.8	27.6	24.2; 31.3
LA and LWA	265	234; 299	8916.8	29.7	26.2; 33.5
LA, SN, and LWA	251	221; 284	8916.8	28.1	24.8; 31.9

a LA: Labour Inspection Authority. SN: Statistics Norway (mortality register). LWA: Labour and Welfare Administration (public compensation).

**Table 3:** Occupational injury deaths in land-based activities among residents of Norway identified in four overlapping registration systems, 2000–2003.

	Total deaths	Labour inspection authority	Statistics Norway	Labour and welfare administration	Finance Norway
Total deaths (%)	246 (100)	171 (70)	141 (57)	158 (64)	50 (*)

Registered in only one data source					
1 source, total	**58**	:	:	:	:
	20	20	–	–	–
	8	–	8	–	–
	25	–	–	25	–
	5	–	–	–	5

Registered in two overlapping data sources					
2 sources, total	**108**	:	:	:	:
	30	30	30	–	–
	39	39	–	39	–
	7	7	–	–	7
	24	–	24	24	–
	6	–	6	–	6
	2	–	–	2	2

Registered in three overlapping data sources					
3 sources, total	**74**	:	:	:	:
	50	50	50	50	–
	12	12	12	–	12
	7	7	–	7	7
	5	–	5	5	5

Registered in four overlapping data sources					
4 sources, total	**6**	:	:	:	:
	6	6	6	6	6

Symbols: * Not calculated because of a lack of comparability with the three other data sources. : Category not applicable. - Nil.

**Fig. 2:**
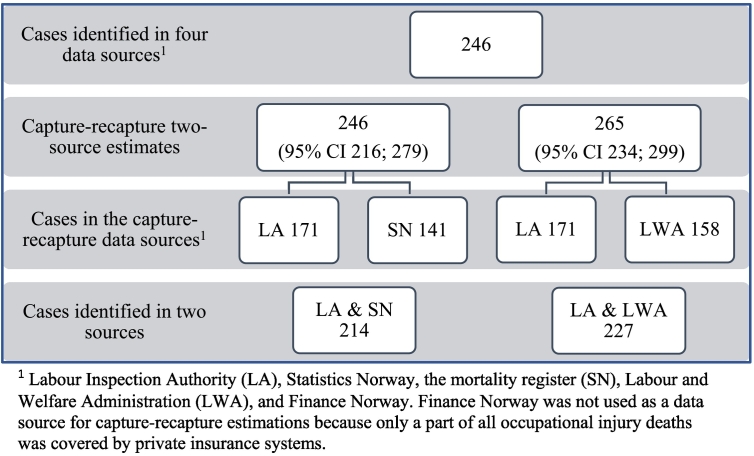
Estimated and observed number of occupational injury deaths among residents of Norway employed in land-based activities, 2000–2003.

The total numbers of occupational injury deaths identified in the four administrative registration systems were the same as one of the two-source capture-recapture estimates: 246 cases (Fig. 2, Table 2, Table 3). The Labour Inspection Authority had registered 70% of the 246 identified cases, and the cause-specific mortality registered 57%. The Labour Inspection Authority had registered 73 fatalities that were not identified as occupational injuries in the cause-specific mortality register in Statistics Norway, and it had 43 cases that were not registered by the Labour Inspection Authority during the four-year period under study. The Labour and Welfare Administration had 64% coverage of the total 246 deaths.

We identified 32 new cases registered by the Labour and Welfare Administration and the insurance companies represented by Finance Norway, cases that were not registered in the two sources we initially examined (Table 3): 25 cases were only registered by the Labour and Welfare Administration, 5 cases were noted only by Finance Norway and 2 cases were registered in both registers.

Based on data from the four registration systems, industry (*n* = 45), agriculture and forestry (*n* = 41), transport and communication (n = 41), and construction (*n* = 27) were the industries with the highest risk of mortality and accounted for 63% of all work injury deaths from 2000 to 2003. The four industries with the highest mortality risk per 100,000 employed persons were mining, agriculture and forestry, transport and communication, and construction (data not shown).

The application of capture-recapture models to three of the data sources (Finance Norway was excluded) showed that a model that allows for source dependency did not give a significantly better model fit than a model based on independency (likelihood ratio statistic 2.66, df = 3, *P*-value = 0.448). The independent model estimated that the number of missing cases (cases not registered by any of the three data sources: the Labour Inspection Authority, Statistics Norway or the Labour and Welfare Administration) was 14 (14.4). The estimate obtained with the model based on source dependency was 10 (10.2) work-related injury deaths.

## Discussion

This study had some important main findings. First, we showed that performing the capture-recapture method on two overlapping registration systems gave reasonable estimates of the true number of work-related injury deaths. In fact, no differences were observed between the two-source estimates (with overlapping confidence intervals) and the actual number of cases identified in the four registration systems (Fig 1). Furthermore, no differences were noted between the two-source and three-source estimates (Table 2). We found several weaknesses in the registration systems, both in the use of definitions (especially of the term “accident”) and in the comparability and quality of the national registers. The Norwegian Directorate of Labour Inspection had the most complete statistics on fatal work injuries in land-based industries among residents, compared to the three other data collection systems. Our assessment gave strong indications that both the administrative registers and the insurance-based systems missed cases. Therefore, fatal work-related injuries were a bigger burden than in governmental statistics. In addition, some of the bereaved may not have received their rightful compensation, although this subject was not within the scope of the study.

### Estimates and actual numbers

The capture-recapture method is useful in estimating the number of cases not registered in any of the data sources. Nevertheless, source dependency (i.e., correlation in capture probability across data sources) is a major factor in the potential bias of capture-recapture estimates. Tilling [24], with reference to Hook and Regal [26], stated that neither of the related assumptions (independency between the data sources and the same case probability to be captured) for using the capture-recapture method ‘*can be directly tested, and violation of either could lead to over- or underestimation of the true population size*’ [24] (p. 12). Underestimation occurs when a “positive” dependency is observed in both data sources, meaning that if a case is captured in one source, it will increase the probability of being captured by both sources [23]. Overestimation occurs in “negative” dependent data sources. In our previous papers [17,18], we did not perform any test of source dependency. For this reason, we assumed that dependency and the probability of being registered (captured) were not the same for all. For example, when the police and the local Labour Inspection Authority are notified after a work-related injury death, we may assume that the physician who examines the deceased is informed and reports the case as a work-related death on the medical death certificate. On the contrary, the physician who issues the death certificate is obligated to report unnatural deaths (e.g., a fatal work injury) to the police authorities [22]. The police investigate whether an unnatural death is related to something criminal or inflicted by another person with the intent to injure or kill. In cases of work-related incidents, the police co-operate with the Labour Inspection Authority regarding violations of the Working Environment Act. It may even be more reasonable to assume that a case is recorded as a work-related injury death by the two registration systems (the register of the Labour Inspection Authority and the mortality register) in cases where the deceased is transported to a forensic institute. The forensic institute is responsible for conducting the medical investigation on behalf of the police and is also responsible for issuing the death certificate. Regarding the Labour Inspection Authority and the Labour and Welfare Administration, we also assumed dependency between the data sources. Nevertheless, and contrary to our presumption, the test applied to the data we used to estimate missing cases indicated no significant dependency between the three register systems.

The multiple-method approach in this study (the capture-recapture method and the identification of cases in the four ongoing registration systems) allowed us to suggest that the capture-recapture estimates were quite accurate and close to the true burden of work-related injury deaths in land-based activities among residents. The capture-recapture estimates (both the two-source and three-source estimates) were similar to the actual numbers of work-related injury deaths identified in the four registration systems. It is unlikely that many more cases occurred among employees who had permanent residences in Norway without being registered by one of the four surveillance or compensation systems we reviewed.

### Occupational injury statistics

There is no reason to believe that underreporting occupational injury deaths is unique for Norway. Studies from other countries have reported similar results, with large variations in the size of underreporting. A comparison of coroners' records with official statistics in Australia [28] from 1989 to 1992 showed that one third of work injury deaths were not covered by official occupational health and safety units or compensation agencies (which were the main sources for the statistics). Statistics from occupational and health authorities covered 35%, while compensation agencies covered 57%. A study in Catalonia, Spain [29], using data from 1994 to1998, showed that the deaths that occurred in the 12-month interim after serious work injuries that were caused by the prior work injuries were missed in occupational injury statistics (*n* = 69 deaths, which increased the total number of work-related injury deaths by 8.2%). Recent studies from upper middle-income countries, including Mexico [30], Brazil [31] and Botswana [32], have indicated that work-related injuries are a much bigger problem than the story that the official statistics tell. An assessment of available data from Southern Africa [33] in the 1990s (mainly insurance and mining/construction company information) showed that a major source of bias in reported data was poor coverage of large sectors of employment (only 20% of the labour force in the region worked in formal-sector wage labour). The most significant share of non-wage labour was in agriculture, and the wage activities not covered were in the retail, repair, and service sectors. Furthermore, most low-status and insecure jobs were often allocated to women, and in some cases, involved children. The reported occupational injury fatality rates in countries in Southern Africa varied from 0.85 to 21.61 per 100,000 workers [33], which indicates a risk level up to 11 times higher than the Labour Inspection Authority's statistics for workers in Norway at the beginning of the 21st century and 7–8 times higher than our adjusted estimates (Fig. 1, Table 2). The potential for prevention is therefore large.

### The term “work accident”

All four register systems used the term “occupational accident” in their definition of occupational injury, but the definitions of “accident” differed. Only one of the systems (Statistics Norway, the mortality register) used the term in accordance with the definition in the International Statistical Classification of Diseases (ICD) (e.g., about injuries or poisonings due to an unintentional incident). The other registries used it as an “umbrella term” and registered violence (homicide) as a work accident, which is in line with the European Statistical Office (Eurostat) in the framework of data collection on European statistics on accidents at work (ESAW) [34,35]. The Labour and Welfare Administration accepted and registered cases of intentional self-harm (suicide) as work accidents, which is in line with the US census for fatal occupational injuries where fatal unintentional injuries and acute poisonings, suicides and homicides are included [36]. Suicide has also been accepted by the courts as a compensable work accident [37], and a study from France suggested a multi-source approach to identifying work-related suicides [38]. Including work-related homicide, assault, suicide and intentional self-harm in the definition of a fatal or non-fatal work injury would reflect all types of occupational injuries independent of the intent; address the responsibility for prevention, compensation, or legal reaction in a correct context; and might give more valid statistics. To compile valid official statistics on fatal and non-fatal work injuries, it is essential that data collection systems use clear and non-interpretable concepts. We therefore suggest a revision of the Norwegian Working Environmental Act, especially Section 5-2 on the employer's notification obligation [20], to make it clearer that the notification is not limited to accidents. Clear definition is key to improving the quality of routine data. Evidence on whether a work injury was an unintentional incident, intentionally inflicted by others or self-inflicted supports different prevention strategies.

### Lethality: proxy indicator of comparability

The ratio between reported fatal and non-fatal work injuries is used as a proxy indicator of the validity and comparability of occupational injury statistics. Applying the indicator across countries may show improbable patterns (e.g., in the formerly socialist countries in Europe) [39]. These countries have reported significantly high mortality rates of work injuries compared with Western Europe (EU-15), but not for non-fatal injuries. The Baltic Sea Network on Occupational Health and Safety (Denmark, Estonia, Finland, Germany, Latvia, Lithuania, Northwest Russia, Norway, Poland and Sweden) developed a method to adjust for underreporting, and in a recent analysis, the research network suggested that the reported level of non-fatal work injuries in several Baltic Sea Network countries was less than 10% to 20% of the estimated true level [39].

Work injuries may not be reported for many reasons, from insecure employment, fear of negative consequences for the one who reports and deliberate attempts to conceal work injuries due to a lack of trust in governmental institutions and poor data collection systems. A recent study, which included data from the International Labour Organization (ILOSTAT) and some other international databases, offered an examination of different factors that may impact the accuracy of national occupational injury statistics, especially the relationship between freedom of the press and the lethality rate (number of work injury fatalities/10,000 total work injuries) [40]. In total, 39 countries were included in the analysis. Adjusting for national indicators, the study showed that ‘*only freedom of the press was associated with the lethality rate per 10,000 injuries in the report of ILOSTAT. The lethality rate of occupational injury reported by each country might not reflect the actual lethality, but under-reported nonfatal occupational injuries, probably relating to freedom of the press*’ [40] (p. 1).

### Limitations

To our knowledge, this is the first study from a Nordic country that used linked surveillance and administrative and compensation data to identify the actual number of occupational injury deaths as a method to validate the capture-recapture estimates of the true numbers. This study has some limitations. First, we might not have identified all cases in the four registers, mainly because of differences in the use of the term “work accident” and variations in the routines around reporting and registration. For example, the mortality register excluded work fatalities due to assault (homicide), and insurance companies (Finance Norway) may have registered some cases under the name of the bereaved who received the compensation, not that of the deceased person. In cases of work traffic accidents, insurance companies may have changed the status from occupational injury compensation to a car insurance case to ensure the best compensation for the injured party (Kari S. Mørk, Finance Norway, personal communication 19 January 2015). Another limitation is that our study covered only land-based activities among the residents of Norway and excluded work-related suicides and non-resident persons working in Norway (Table 1). Therefore, the total burden of fatal work injuries in Norway was higher than our estimates.

Additionally, the data we used are nearly 20 years old, and it may be questioned whether the results are a valid representation of the present state of occupational injury data. The Labour Inspection Authority has modified the reporting system since 2004 regarding technical solutions, data elements and organization. Furthermore, the ability to retrieve unreported cases may have increased: the Labour Inspection Authority cooperates closely with the police authority on reporting serious occupational injuries, receives reports from emergency medical centres and uses the Internet to detect reports about work-related injury deaths in the media [41,42]. The Labour Inspection Authority has suggested that the improved reporting systems may partly explain why the mortality rates of work-related injuries in Norway did not decrease from 2001 to 2014 [41]. In a more recent report from the Labour Inspection Authority, it was assumed that the registration is now fairly complete and that the observed decline in fatal occupational injury rates from 2010 to 2019 is real [42]. However, we do not know whether the modifications in the registration system since 2004 have affected the completeness and quality of the data and the comparability of time trends. A new project has therefore been planned and initiated.

## Conclusion

The occupational injury rate was 27.6 per 1 million employed persons (based on all cases identified in the four registers), corresponding to more than one fatal work injury every week on average in land-based activities in Norway (in the beginning of the 21 century). The actual number of fatal work injuries (246) was 44% higher than the number registered by the Labour Inspection Authority. The study showed that an insufficient quality of routine data is a problem, also in high-income countries such as Norway. Capture-recapture estimates based on two sources may give a more realistic picture of the burden of fatal occupational injuries. A new project is planned to assess whether completeness improved after 2003.

## Funding

This research did not receive any specific grants from funding agencies in the public, commercial or not-for-profit sectors.

## Declaration of Competing Interest

The authors declare that they have no known competing financial interests or personal relationships that could have appeared to influence the work reported in this paper.
